# Prognostic value of N-terminal Pro–B-Type natriuretic peptide in patients with intermediate coronary lesions

**DOI:** 10.3389/fcvm.2022.903757

**Published:** 2022-07-28

**Authors:** Chenxi Song, Sheng Yuan, Kongyong Cui, Zhongxing Cai, Rui Zhang, Jining He, Zheng Qiao, Xiaohui Bian, Shaoyu Wu, Haoyu Wang, Rui Fu, Chunyue Wang, Qianqian Liu, Dong Yin, Lei Jia, Kefei Dou

**Affiliations:** ^1^Cardiometabolic Medicine Center, Department of Cardiology, Fuwai Hospital, National Center for Cardiovascular Diseases, Chinese Academy of Medical Sciences and Peking Union Medical College, Beijing, China; ^2^State Key Laboratory of Cardiovascular Disease, Beijing, China

**Keywords:** coronary artery disease, intermediate coronary lesions, NT-proBNP, MACE, biomarker

## Abstract

**Background:**

The optimal treatment strategy for patients with coronary intermediate lesions, defined as diameter stenosis of 50–70%, remains a great challenge for cardiologists. Identification of potential biomarkers predictive of major adverse cardiovascular events (MACEs) risk may assist in risk stratification and clinical decision.

**Methods:**

A total of 1,187 patients with intermediate coronary lesions and available N-terminal pro-brain natriuretic peptide (NT-proBNP) levels were enrolled in the current study. A baseline NT-proBNP level was obtained. The primary endpoint was defined as MACEs, the composite endpoint of all-cause death and non-fatal myocardial infarction. A multivariate Cox regression model was used to explore the association between NT-proBNP level and MACE risk.

**Results:**

The mean age of the study cohort was 59.2 years. A total of 68 patients experienced MACE during a median follow-up of 6.1 years. Restricted cubic spline analysis delineated a linear relationship between the baseline NT-proBNP level and MACE risk. Both univariate and multivariate analyses demonstrated that an increased NT-proBNP level was associated with an increased risk of MACE [adjusted hazard ratio (*HR*) per doubling: 1.412, 95% confidence interval (*CI*): 1.022–1.952, *p* = 0.0365]. This association remains consistent in clinical meaningful subgroups according to age, sex, body mass index (BMI), and diabetes.

**Conclusion:**

An increased NT-proBNP level is associated with an increased risk of MACE in patients with intermediate coronary lesions and may serve as the potential biomarker for risk stratification and treatment decision guidance.

## Introduction

Coronary artery disease (CAD) remains a major burden of mortality and morbidity globally (1). Intermediate severity coronary lesions, defined as diameter stenosis between 50–70%, account for more than 20% of patients receiving coronary angiography (2). Despite the advance in invasive coronary functional assessment, the optimal strategy and prognosis for patients with intermediate coronary lesions still need investigation. In addition to the discussion of optimal invasive angiography methods, specific predictors for patients with intermediate severity coronary lesions may contribute to the risk stratification and clinical decision.

An N-terminal pro-brain natriuretic peptide (NT-proBNP) is a classic biomarker of heart failure (HF) (3). In addition, many studies have revealed its strong correlation with the prognosis of ischemic heart diseases, pulmonary embolism, and other cardiovascular diseases (4–6). A cohort study consisting of 216 patients with acute myocardial infarction (AMI) found that patients with increased NT-proBNP (> 183 ng/L) were at a higher risk of death or HF in the 8 years after acute coronary syndrome (7). Meanwhile, the baseline NT-proBNP concentration has been prospectively proved to be associated with 2-year cardiovascular events in patients with stable angina, which was independent of hsCRP (LVEF) (8). Similarly, a 9-year follow-up of patients with stable CAD identified NT-proBNP as a strong prognostic marker beyond traditional cardiovascular risk factors (9). However, there is little evidence about the prognostic value of NT-proBNP in intermediate coronary lesions.

Intermediate coronary lesions are at the middle stage of the progression of CAD. With the advances in invasive coronary technologies, such as fractional flow reserve (FFR), current guidelines recommend a safe deferral of revascularization for intermediate lesions without functional ischemia (FFR > 0.80 or 0.75) (10). However, a randomized clinical trial has found that patients with intermediate coronary lesions who received conservative treatment (optimal pharmacologic therapy) still had a 7.3% rate of the primary endpoint (a composite of all-cause death, any myocardial infarction, or any revascularization at 1 year) (11), which suggested that angiography alone may not be competent for the risk stratification. More comprehensive information including prognostic indicators will facilitate optimal clinical decision making. Therefore, our study aims to investigate the prognostic value of NT-proBNP in patients with intermediate coronary lesions.

## Materials and methods

### Participants

Consecutive patients who underwent coronary angiography in the year 2013 were prospectively enrolled from Fuwai hospital. Eligible patients had at least one lesion with an angiographic stenosis of 50–70%. We excluded patients who had lesions with stenosis greater than 70%, with a history of percutaneous coronary intervention (PCI) or coronary artery bypass graft surgery (CABG), underwent PCI or CABG revascularization during hospitalization, or without available data on NT-proBNP. In addition, we excluded those who lost-to follow-up. The study protocol complied with the principles of the Declaration of Helsinki and was approved by the Review Board of Fuwai Hospital. Written informed consent was obtained from each participant.

### Exposure and outcome

The plasma NT-proBNP level was determined by using the sandwich enzyme immunoassay (BI-20852W, Biomedica, Vienna, Austria). A fasting blood sample was collected on admission prior to angiography. The blood samples were collected into EDTA-anticoagulant tubes and centrifuged to obtain the plasma, which was used to determine the NT-proBNP level. Follow-up was performed by trained cardiologists *via* telephone call or clinical visit around the year 2019. All events were carefully verified by an independent group of clinical physicians. The primary outcome was defined as MACE, which was a composite endpoint of all-cause death and non-fatal myocardial infarction.

### Statistical analysis

Continuous data were presented as mean ± SD or median (interquartile), and compared by using analysis of variance or the Mann–Whitney *U*-test. Categorical variables were presented as frequency (percentage) and compared with the chi-square test or Fisher’s exact test as appropriate. Restricted cubic spline was used to flexible the model and characterize the relationship between NT-proBNP and MACE.

The Schoenfeld Residuals Test was carried out to test the proportional hazard assumption in the Cox regression model. Univariate Cox proportional hazard regressions (*HR*s) were performed to explore the association between each variable and outcome, and the *HR* [95% confidence interval (*CI*)] was calculated for each variable. A multivariate Cox proportional hazard regression model was used to explore the association between NT-proBNP and outcome. A total of three models was used to increase the robustness of our results. Model 1 adjusted for age, sex, body mass index (BMI); Model 2 adjusted for model 1 + smoking status, hypertension, diabetes, left ventricular ejection fraction (LVEF) < 50%; Model 3 adjusted for model 2 + high-sensitivity C-reactive protein (hsCRP), endothelin, Hemoglobin A1C (HbA1c), and Free triiodothyronine (FT_3_). Due to the limited number of cases, covariates were carefully chosen based on their clinical and statistical significance. Covariates included in the multivariate model had a *p*-value < 0.05 in univariate analysis or were clinically judged to be important prognostic factors in the setting of CAD. We performed collinearity analysis by calculating the variance inflation factor (VIF) to explore variables that were significantly associated with the NT-proBNP. Scatter plots between NT-proBNP and ejection fraction (EF)/creatine were plotted to visualize the relationship between extremely high levels of NT-proBNP and HF/renal function. After excluding patients with NT-proBNP values greater than the 99th percentile, a sensitivity analysis was conducted to evaluate the impact of extremely high NT-proBNP on the association between NT-proBNP and MACE risk. A subgroup analysis was performed to investigate whether the association between NT-proBNP level and MACE differed by subgroup according to age, sex, BMI, and diabetes. The statistical analysis was performed by SAS software Version (SAS Institute, United States) and R version 4.0.2 (R Foundation for Statistical Computing, Vienna, Austria).

## Results

### Baseline characteristics

**FIGURE 1:**
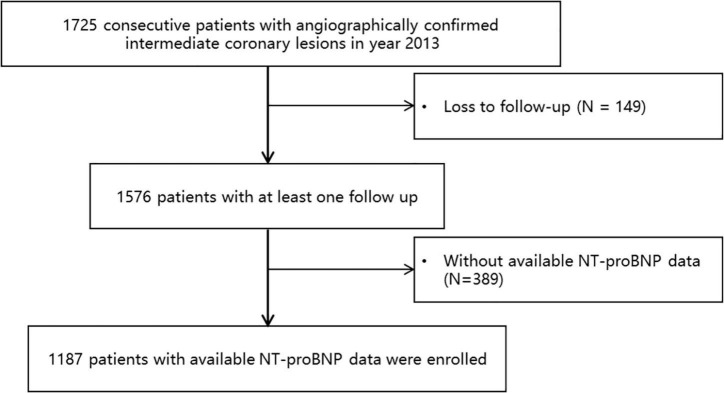
A study flowchart. A total of 1,725 consecutive patients who underwent coronary angiography in the Fuwai hospital in the year 2013 with angiographically confirmed intermediate coronary lesions were enrolled. Patients loss to follow-up or with unavailable N-terminal pro-brain natriuretic peptide (NT-proBNP) data were excluded. A total of 1,187 patients were finally included in the current analysis.

Of the 1,187 participants enrolled in our study (Figure 1), 375 (31.59%) were women with a median age of 59.20 years. The median follow-up time was 6.13 years [interquartile range (IQR): 5.79–6.68 years]. The median NT-proBNP level was 544.70 pmol/L [interquartile range (IQR): 431.40–718.30 pmol/L]. There was no significant difference of baseline characteristics between patients with and without follow-up records (Supplementary Table 1). Baseline characteristics according to the baseline NT-proBNP level are summarized in Table 1. The median age increased progressively across NT-proBNP tertiles, and the median age was greater in patients with NT-proBNP level in the third tertile compared with the first or second tertile. No significant difference in the past medical history, such as hyperlipidemia, diabetes, smoking status, and alcohol consumption were found across these three groups. LVEF decreased significantly across tertiles. The level of hsCRP and creatinine level increased progressively across NT-proBNP tertiles. Patients with an admission NT-proBNP level in the third tertile had higher total cholesterol, low low-density lipoprotein cholesterol (LDL-c), and high-density lipoprotein cholesterol (HDL-c) level compared with the first or second tertile. The proportion of left anterior descending (LAD) lesion was lower in the third tertile compared with the first or second tertile. The proportion of the use of clopidogrel and nitrates was lower in the third tertile compared with the first or second tertile.

**TABLE 1:** Baseline characteristics between patients according to N-terminal pro-brain natriuretic peptide (NT-proBNP) tertile.

	Overall *N* = 1187	1^st^ tertile *N* = 396	2^nd^ tertile *N* = 395	3^nd^ tertile *N* = 396	*P*-value
NT-proBNP (pmol/L)	34.40–9388.50	34.40–472.40	472.60–636.70	636.80–9388.50	
Age (years)	59.20 (52.25, 65.90)	57.95 (50.98, 64.23)	58.20 (52.20, 64.80)	61.75 (55.75, 68.43)	< 0.001
Female (%)	375 (31.59)	121 (30.56)	113 (28.61)	141 (35.61)	0.092
AMI (%)	36 (3.03)	8 (2.02)	8 (2.03)	20 (5.05)	0.016
Hypertension (%)	780 (65.71)	247 (62.37)	283 (71.65)	250 (63.13)	0.010
Diabetes (%)	266 (22.41)	86 (21.72)	89 (22.53)	91 (22.98)	0.911
Hyperlipidemia (%)	704 (59.31)	238 (60.10)	235 (59.49)	231 (58.33)	0.876
Smoke (%)	515 (43.39)	166 (41.92)	187 (47.34)	162 (40.91)	0.146
Alcohol (%)	543 (45.75)	182 (45.96)	195 (49.37)	166 (41.92)	0.109
BMI (kg/m^2^)	25.59 (23.67, 27.68)	25.60 (23.77, 27.68)	25.78 (24.09, 28.05)	25.26 (23.44, 27.17)	0.021
LVEF (%)	64.88 ± 6.38	65.65 ± 5.18	65.16 ± 5.53	63.76 ± 8.03	0.002
TNI (IU/L)	11.00 (9.00, 13.00)	10.00 (8.00, 13.00)	10.00 (9.00, 13.00)	11.00 (9.00, 14.00)	0.005
hsCRP (mg/L)	1.26 (0.65, 2.62)	1.07 (0.56, 1.91)	1.24 (0.63, 2.57)	1.54 (0.78, 3.43)	< 0.001
Creatinine (μmol/L)	73.19 (63.89, 82.35)	71.39 (62.19, 80.12)	73.30 (63.08, 81.36)	74.19 (64.90, 84.49)	0.0350
D_Dimer (ug/ml)	0.28 (0.19, 0.38)	0.26 (0.18, 0.35)	0.26 (0.19, 0.37)	0.31 (0.21, 0.43)	< 0.001
ESR (mm/h)	6.00 (2.00, 12.00)	6.00 (2.00, 12.00)	6.00 (2.00, 11.00)	7.00 (3.00, 16.00)	0.010
NEU (%)	60.10 (54.50, 65.30)	59.00 (53.58, 64.23)	60.95 (54.85, 65.60)	60.70 (54.90, 66.93)	0.010
TC (mmol/L)	4.14 (3.49, 4.83)	4.09 (3.45, 4.77)	4.08 (3.46, 4.69)	4.26 (3.59, 4.97)	0.013
LDL-c (mmol/L)	2.40 (1.85, 3.03)	2.38 (1.87, 2.98)	2.35 (1.81, 2.92)	2.49 (1.92, 3.20)	0.011
HDL-c (mmol/L)	1.07 (0.90, 1.28)	1.05 (0.87, 1.24)	1.05 (0.90, 1.27)	1.10 (0.92, 1.32)	0.008
Lpa (mg/L)	149.63 (59.70, 329.13)	145.51 (61.34, 306.56)	136.20 (53.02, 335.38)	167.59 (67.56, 353.19)	0.138
Fasting glucose (mg/L)	5.34 (4.87 6.10	5.25 (4.76, 6.11)	5.38 (4.89, 6.16)	5.41 (4.95, 6.04)	0.033
HbA1c (%)	6.10 (5.80, 6.60)	6.10 (5.80, 6.60)	6.10 (5.80, 6.70)	6.20 (5.80, 6.60)	0.324
Endotheline (pmol/L)	0.24 (0.19, 0.30)	0.24 (0.20, 0.29)	0.23 (0.19, 0.30)	0.24 (0.19, 0.33)	0.140
Free T_3_ (pg/mL)	2.96 (2.74, 3.19)	2.96 (2.78, 3.20)	2.99 (2.74, 3.20)	2.93 (2.71, 3.17)	0.128
Free T_4_ (pg/mL)	1.13 (1.03, 1.25)	1.11 (1.02, 1.22)	1.13 (1.03, 1.25)	1.15 (1.04, 1.26)	0.044
TT3 (ng/mL)	1.05 (0.92, 1.20)	1.02 (0.89, 1.17)	1.07 (0.93, 1.22)	1.07 (0.95, 1.22)	0.003
TT4 (μg/mL)	8.50 (7.30, 9.60)	8.15 (7.10, 9.30)	8.70 (7.40, 9.90)	8.60 (7.50, 9.70)	0.002
TSH (μIU/mL)	1.81 (1.21, 2.73)	1.86 (1.21, 2.73)	1.65 (1.10, 2.49)	1.91 (1.28, 2.99)	0.005
**Angiographic characteristics**					
LM (%)	45 (3.79)	13 (3.28)	16 (4.05)	16 (4.04)	0.810
RCA (%)	265 (22.33)	86 (21.72)	89 (22.53)	90 (22.73)	0.937
LAD (%)	768 (64.70)	269 (67.93)	264 (66.84)	235 (59.34)	0.023
LCX (%)	332 (27.97)	94 (23.74)	121 (30.63)	117 (29.55)	0.067
Three-vessel disease	41 (3.45)	13 (3.28)	15 (3.80)	13 (3.28)	0.901
**Medications during hospitalization**					
Clopidogrel (%)	1012 (85.26)	348 (87.88)	346 (87.59)	318 (80.30)	0.003
Heparin (%)	70 (5.90)	10 (2.53)	21 (5.32)	39 (9.85)	< 0.001
Statins (%)	1078 (90.82)	366 (92.42)	364 (92.15)	348 (87.88)	0.046
Beta-blockers (%)	995 (83.82)	335 (84.60)	334 (84.56)	326 (82.32)	0.610
CCB (%)	690 (58.13)	236 (59.60)	228 (57.72)	226 (57.07)	0.756
Nitrates (%)	1101 (92.75)	377 (95.20)	372 (94.18)	352 (88.89)	0.001
Diabetes medicines (%)	234 (19.71)	82 (20.71)	74 (18.73)	78 (19.70)	0.784

AMI, acute myocardial infarction; BMI, body mass index; LVEF, left ventricular ejection fraction; TNI, troponin; hsCRP, high-sensitivity C-reactive protein; ESR, erythrocyte sedimentation rate; NEU, Neutrophil; TC, Total cholesterol; LDL-c, low-density lipoprotein cholesterol; HDL-c, high-density lipoprotein cholesterol; Lpa, Lipoprotein(a); HbA1c, Hemoglobin A1C; Free T_3_, Free triiodothyronine; Free T_4_, Free Thyroxine; TT_3_, Total triiodothyronine; TT_4_, Total Thyroxine; TSH, Thyroid-Stimulating Hormone; LM, left main; RCA, right coronary artery; LAD, left anterior descending; LCX, left circumflex artery; CCB, Calcium channel blockers.

### The association between N-terminal pro-brain natriuretic peptide level and major adverse cardiovascular event

**FIGURE 2:**
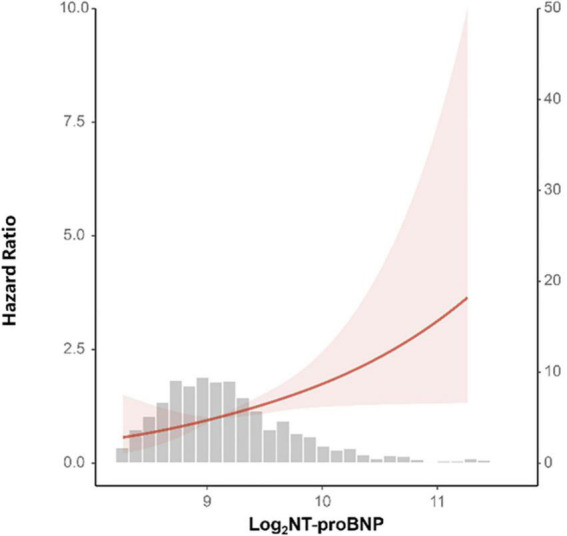
A restricted cubic spline analysis of the association between baseline NT-proBNP level and major cardiovascular event (MACE) risk. Baseline log_2_ transformed NT-proBNP level presented a linear relationship with the risk of MACE (*p* for non-linearity = 0.891). The curves are presented with 95% confidence interval (*CI*).

**FIGURE 3:**
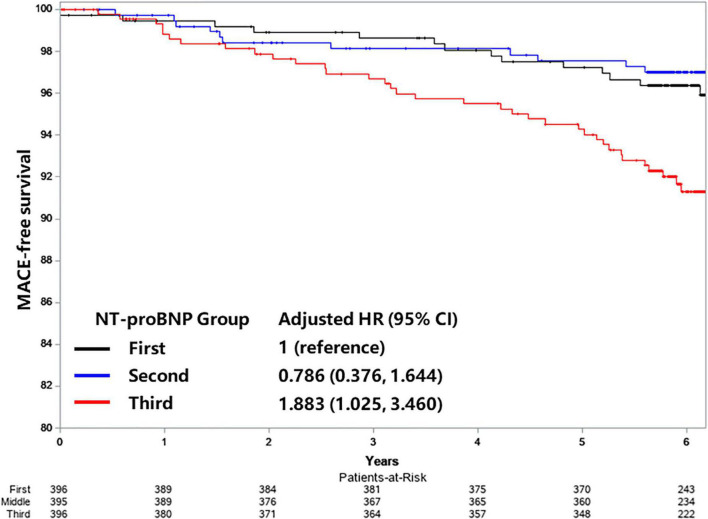
Kaplan–Meier curves of MACE-free survival according to NT-proBNP tertiles. Adjusted for age, sex, body mass index (BMI), smoking status, prior history of diabetes, hypertension, left ventricular ejection fraction (LVEF), the plasma level of high-sensitivity C-reactive protein (hsCRP), endothelin, Hemoglobin A1C (HbA1C), and Free triiodothyronine (FT3).

A total of 68 patients experienced MACE during a median follow-up of 6.1 years (IQR range 5.8–6.8 years). Patients with MACE had higher baseline NT-proBNP compared with patients without MACE [680.15 (486.85, 921.50) vs. 542.00 (430.30, 700.15), *p* = 0.001]. A restricted cubic spline was used to delineate the association between the baseline NT-proBNP level and MACE (Figure 2). NT-proBNP presented a linear relationship with the risk of MACE (*p* for non-linearity = 0.891), and the risk of MACE increased along with NT-proBNP levels. Univariate analysis results are shown in Table 2. An increased NT-proBNP level, when modeled as a continuous variable, was associated with the higher risk of MACE (*HR* per doubling: 1.659, 95% *CI*: 1.268–2.170, *p* = 0.0002). In multivariate analysis, covariates included in the multivariate model were carefully chosen based on clinical significance and the association with MACE due to the limited number of cases (Supplementary Table 2). After adjustment of age, sex, and BMI (model 1), an increased NT-proBNP level, as a continuous variable, was associated with a higher MACE risk (adjusted *HR* per doubling: 1.499, 95% *CI*: 1.138–1.976, *p* = 0.0040) (Table 3). When NT-proBNP level was modeled as a categorical variable, the third NT-proBNP tertile was associated with higher MACE risk compared with the first tertile (adjusted *HR*: 2.096, 95% *CI*: 1.162–3.781, *p* = 0.0140). Further adjustment of smoking status, hypertension, diabetes, LVEF, hsCRP, endothelin, HbA1c, and Free T_3_ did not significantly alter this association. The results of collinearity analysis found no variable that was significantly related to NT-proBNP (Supplementary Table 3). Kaplan–Meier curves of MACE-free survival are shown in Figure 3. Only a few patients had extremely high levels of NT-proBNP while their creatine and EF were normal (Supplementary Figure 1). After excluding patients with extremely high NT-proBNP levels (> 3,188.3 pmol/L), the association was still robust in the model 3 (adjusted *HR* per doubling: 1.521, 95% *CI*: 1.041–2.222, *p* = 0.0300). All Cox models satisfy the proportional hazard assumption, which were estimated by the Schoenfeld Residuals Test (Supplementary Figure 2).

**TABLE 2:** A univariate analysis of the association between NT-proBNP level and major adverse cardiovascular event (MACE) risk.

	Event (%)	HR (95% CI)	*P*-value
NT-proBNP ^a^ (per doubling in value)		1.659 (1.268, 2.170)	0.0002
NT-proBNP as categorical variable			
NT-proBNP ≤ 472.4	16/396 (4.04)	1 (reference)	1 (reference)
472.4 < NT-proBNP ≤ 636.7	13/395 (3.29)	0.826 (0.398, 1.718)	0.6096
NT-proBNP > 636.7	39/396 (9.85)	2.531 (1.414, 4.529)	0.0018

^a^NT-proBNP level was log_2_ transformed.

**TABLE 3:** The multivariable analysis of the association between NT-proBNP level and MACE risk.

	Event (%)	HR (95% CI)	*P*-value
**NT-proBNP ^a^ (per doubling in value)**			
Model 1		1.499 (1.138, 1.976)	0.0040
Model 2		1.471 (1.100, 1.967)	0.0092
Model 3		1.412 (1.022, 1.952)	0.0365
**NT-proBNP as categorical variable**			
**Model 1**			
NT-proBNP ≤ 472.4	16/396 (4.04)	1 (reference)	1 (reference)
472.4 < NT-proBNP ≤ 636.7	13/395 (3.29)	0.814 (0.391, 1.692)	0.5807
NT-proBNP > 636.7	39/396 (9.85)	2.096 (1.162, 3.781)	0.0140
**Model 2**			
NT-proBNP ≤ 472.4	16/396 (4.04)	1 (reference)	1 (reference)
472.4 < NT-proBNP ≤ 636.7	13/395 (3.29)	0.795 (0.382, 1.656)	0.5403
NT-proBNP > 636.7	39/397 (9.85)	2.005 (1.107, 3.634)	0.0217
**Model 3**			
NT-proBNP ≤ 472.4	16/396 (4.04)	1 (reference)	1 (reference)
472.4 < NT-proBNP ≤ 636.7	13/395 (3.29)	0.786 (0.376, 1.644)	0.5232
NT-proBNP > 636.7	39/397 (9.85)	1.883 (1.025, 3.460)	0.0415

Model 1 adjusted for age, sex and BMI; Model 2 adjusted for model 1 + smoking status, hypertension, diabetes, and LVEF; Model 3 adjusted for model 2 + hsCRP, endothelin, HbA1c, and Free T_3_.

### Subgroup analysis

**FIGURE 4:**
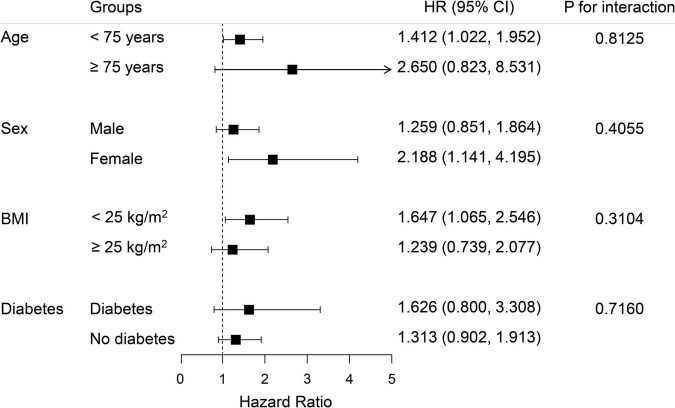
The subgroup analysis of the association between NT-proBNP level and MACE risk according to age, sex, BMI, and diabetes. Adjusted for age, sex, BMI, smoking status, prior history of diabetes, hypertension, LVEF, plasma level of hsCRP, endothelin HbA1C, and Free T3.

Subgroup analysis was performed to explore whether the association between NT-proBNP and MACE risk differed in clinical meaningful subgroups (Table 4). In general, no significant interaction was observed between the NT-proBNP level and age, sex, BMI, and diabetes on MACE risk (*p*-value for interaction was greater than 0.05 for all subgroups). In each age subgroup (age < 75 years or age ≥ 75 years), female or male subgroup, obese or non-obese subgroup (BMI < 25 kg/m^2^ or BMI ≥ 25 kg/m^2^), diabetes or no diabetes subgroup, increased NT-proBNP level was associated with a higher risk of MACE. The forest plot is shown in Figure 4.

**TABLE 4:** The subgroup analysis of the association between NT-proBNP and MACE.

	MACE rate (%)	HR (95% CI)	*P*_*interaction*_
Age			0.8125
< 75 years (*N* = 1119)	59/1119 (5.27)	1.412 (1.022, 1.952)	
≥ 75 years (*N* = 465)	9/68 (13.24)	2.650 (0.823, 8.531)	
Sex			0.4055
Female (*N* = 375)	18/375 (4.80)	2.188 (1.141, 4.195)	
Male (*N* = 812)	50/812 (6.16)	1.259 (0.851, 1.864)	
BMI (kg/m^2^)			0.3104
< 25 kg/m^2^ (*N* = 510)	32/510 (6.27)	1.647 (1.065, 2.546)	
≥ 25 kg/m^2^ (*N* = 67)	36/677 (5.32)	1.239 (0.739, 2.077)	
Diabetes			0.7160
With Diabetes	48/921 (5.21)	1.626 (0.800, 3.308)	
No diabetes	20/266 (7.52)	1.313 (0.902, 1.913)	

Adjusted for age, sex, BMI, smoking status, prior history of diabetes, hypertension, LVEF, plasma level of hsCRP, endothelin HbA1C and Free T_3_. NT-proBNP level was log_2_ transformed.

## Discussion

### Major findings

The current study enrolled a total of 1,187 patients with intermediate coronary lesions, in which PCI was deferred. Increased NT-proBNP level, both modeled as a continuous or categorical variables, was associated with higher MACE risk at a median 6-year follow-up after the adjustment of demographic variables, LVEF, cardiovascular risk factors, and other biomarker levels. This association was consistently observed according to clinical meaningful subgroups according to age, sex, BMI, and diabetes.

### Mechanisms underlying elevated N-terminal pro-brain natriuretic peptide in stable coronary artery disease

Brain natriuretic peptide/NT-proBNP is synthesized in response to increased end-diastolic wall stress, in the setting of increased intracardiac volumes and filling pressures, and acts to natriuresis, diuresis, vasodilatation, improved myocardial relaxation, and reduced myocardial fibrosis *via* binding to multiple NP receptors (12). BNP and NT-proBNP are the fundamental biomarkers used for the diagnosis and prognosis evaluation in the setting of HF (3). Since natriuretic peptides are secreted from the ventricle in response to volume or pressure overload, elevations in the NT-proBNP level reflect adverse hemodynamic alterations and subclinical ventricular dysfunction. Consistently, there is a negative association between NT-proBNP level and LVEF (9), which is the most commonly used echo index used for evaluating cardiac systolic function. Reduced left ventricular function as reflected by LVEF is associated with a worse outcome in patients with CAD (13). Thus, the association between increased NT-proBNP levels and an increased risk of MACE can be partly explained by the impaired cardiac function in patients with the increased cardiac function.

Recent studies showed that myocardial ischemia increased the BNP gene expression and plasma BNP levels (14). Increased levels of BNP are independently associated with inducible ischemia by exercise treadmill testing among outpatients with stable coronary disease (15). Thus, NT-proBNP may be an indicator for myocardial ischemia and associated with reduced event-free survival.

### Comparison with previous studies

The present study extends previous studies by providing evidence supporting the prognostic role of NT-proBNP in patients with intermediate coronary lesions. Previous studies have demonstrated that an increased NT-proBNP level was predictive of the long-term MACE risk in stable patients with CAD (16, 17). However, over half of the patients have lesions with a narrowing of the lumen by more than 70% (9), or underwent revascularization in those studies (9, 18, 19), and whether the findings can be generalized to patients with angiographic intermediate lesions who did not receive revascularization remained unclear.

### Clinical significance

Our findings suggest NT-proBNP as a novel biomarker for risk stratification and identify the high-risk population who may benefit from intensive treatment, such as intensive lipid lowering therapy or revascularization. FFR is the “gold-standard” for the assessment of intermediate coronary lesions, and is recommended by current guidelines (20, 21), however, FFR is largely underused in clinical practice due to the invasiveness of the procedure, additional procedure time and cost, and potential adverse effects of the hyperemic agents. The level of NT-proBNP may be increased in the setting of subclinical cardiac dysfunction and myocardial ischemia, and serve as a novel biomarker to identify those patients at high risk of adverse events.

### Limitations

Our findings are subject to limitations of observational cohort studies. Residual and unmeasured confounders could not be excluded. The sample size of our study was moderate, and our findings require further validation in larger-scale prospective cohort study. Second, physiological measurement of intermediate lesions, which is recommended for the assessment of functional significance of lesions and the guidance of PCI strategy, was not available for this study. Third, in addition to LVEF, other echo-indices used for the assessment of cardiac function were not available. Fourth, the current study excluded participants who underwent revascularization previously or during hospitalization. The prognostic value of NT-proBNP value in patients with intermediate lesions in which PCI treatment was performed requires future investigation. Fifth, beta-blockers, angiotensin receptor-neprilysin inhibitors (ARNi), angiotensin-converting-enzyme inhibitors, and diuretics can all impact NT-proBNP levels. However, due to data collection limitations, drug information in this database was limited, and we could only compare the proportion of beta-blockers. Sixth, due to a limitation of follow-up data, we could only compare the results of all-cause death and non-fatal myocardial infarction; more endpoints, such as stroke and rehospitalization for HF, should be highlighted in future research. Finally, only baseline NT-proBNP level were measured. The association between serial NT-proBNP level change and mortality requires future exploration.

## Conclusion

N-terminal pro-brain natriuretic peptide is associated with MACE risk in patients with intermediate coronary lesions. This association was consistently observed in clinical meaningful subgroups according to age, sex, BMI, and diabetes. The NT-proBNP level may serve as the potential biomarker for risk stratification and treatment decision guidance in the setting of coronary intermediate lesions.

## Data availability statement

The original contributions presented in this study are included in the article/Supplementary material, further inquiries can be directed to the corresponding authors.

## Ethics statement

The studies involving human participants were reviewed and approved by the Review Board of Fuwai Hospital. The patients/participants provided their written informed consent to participate in this study.

## Authors contributions

KD and LJ: conception, design, final approval of the article, and overall responsibility. CS, KC, SW, HW, RF, CW, and SY: data collection. SY, ZC, QL, and RZ: analysis and interpretation. JH, RZ, and CS: article writing. ZQ and XB: critical revision. KD and DY: obtain funding. All authors contributed to the article and approved the submitted version.
