# Supplementary material for: Prognostic value of N-terminal Pro–B-Type natriuretic peptide in patients with intermediate coronary lesions
Source: Front Cardiovasc Med. 2022 Jul 28;9:903757. doi: 10.3389/fcvm.2022.903757 (PMC9370998; doi:10.3389/fcvm.2022.903757)
Supplement: Supplementary file 1 [file Data_Sheet_1.pdf]

Supplementary Material

1 TableS1. Comparison of Baseline Characteristics between patients with and without follow-up records

	Loss to follow-up N = 149	With follow-up records N = 1576	P value
NT-proBNP (pmol/L)	540.10 [445.15, 687.90]	544.70 [431.45, 717.85]	0.999
Age (years)	58.60 [51.80, 65.40]	59.10 [52.70, 65.60]	0.541
Female (%)	53 (35.57)	503 (31.92)	0.412
AMI (%)	1 (0.67)	61 (3.87)	0.076
Hypertension (%)	95 (64.63)	1031 (65.42)	0.918
Diabetes (%)	29 (19.73)	348 (22.08)	0.578
Hyperlipidemia (%)	83 (55.70)	932 (59.14)	0.467
Smoke (%)	57 (38.78)	689 (43.72)	0.285
Alcohol (%)	62 (42.18)	708 (44.92)	0.58
BMI (kg/m^2)	25.37 [23.44, 27.31]	25.58 [23.62, 27.68]	0.618
LVEF (%)	66.00 [63.00, 70.00]	65.00 [62.00, 68.00]	0.092
TNI (IU/L)	11.00 [9.00, 14.00]	10.00 [9.00, 13.00]	0.046
hsCRP (mg/L)	1.38 [0.69, 2.32]	1.29 [0.67, 2.65]	0.71
Creatinine (umol/L)	74.18 [64.10, 83.93]	73.01 [63.12, 82.03]	0.459
D_Dimer (ug/ml)	0.29 [0.20, 0.38]	0.28 [0.19, 0.38]	0.424
ESR (mm/h)	7.00 [3.00, 12.00]	6.00 [3.00, 12.00]	0.984
NEU (%)	59.55 [53.92, 67.80]	60.00 [54.00, 65.40]	0.413
TC (mmol/L)	4.25 [3.68, 5.00]	4.11 [3.46, 4.83]	0.161
LDL-c (mmol/L)	2.39 [1.95, 3.02]	2.39 [1.83, 3.03]	0.399
HDL-c (mmol/L)	1.06 [0.89, 1.28]	1.05 [0.88, 1.27]	0.71
Lpa (mg/L)	150.42 [70.73, 318.95]	151.46 [60.10, 333.94]	0.935
Fasting glucose (mg/L)	5.38 [4.92, 6.11]	5.37 [4.88, 6.16]	0.998
HbA1c (%)	6.00 [5.70, 6.40]	6.10 [5.80, 6.60]	0.08
Endotheline (pmol/L)	0.24 [0.18, 0.31]	0.24 [0.20, 0.31]	0.407
Free T_3 (pg/mL)	2.97 [2.75, 3.23]	2.96 [2.74, 3.19]	0.565
Free T_4 (pg/mL)	1.15 [1.04, 1.23]	1.12 [1.02, 1.24]	0.594
TT3 (ng/mL)	1.06 [0.93, 1.18]	1.04 [0.91, 1.19]	0.521
TT4 (ug/mL)	8.50 [7.50, 9.60]	8.40 [7.20, 9.50]	0.301
TSH (uIU/mL)	1.81 [1.15, 2.93]	1.84 [1.21, 2.77]	0.564
Angiographic characteristics			
LM (%)	6 (4.03)	56 (3.55)	0.947
RCA (%)	37 (24.83)	357 (22.65)	0.614
LAD (%)	97 (65.10)	1015 (64.40)	0.936
LCX (%)	35 (23.49)	439 (27.86)	0.296
Three-vessel disease	4 (2.68)	55 (3.49)	0.779
Medications during hospitalization			
Clopidogrel (%)	131 (89.12)	1333 (84.58)	0.177
Heparin (%)	7 (4.76)	102 (6.47)	0.524

Statins (%)	128 (87.07)	1430 (90.74)	0.195
Beta-blockers (%)	121 (82.31)	1316 (83.50)	0.799
CCB (%)	90 (61.22)	917 (58.19)	0.53
Nitrates (%)	136 (92.52)	1462 (92.77)	1
Diabetes medicines (%)	22 (14.97)	296 (18.78)	0.303

AMI= acute myocardial infarction; BMI = body mass index; LVEF = left ventricular ejection fraction; TNI = troponin; hsCRP=high-sensitivity C-reactive protein; ESR = erythrocyte sedimentation rate; NEU = Neutrophil; TC = Total cholesterol; LDL-c = low-density lipoprotein cholesterol; HDL-c = high-density lipoprotein cholesterol; Lpa = Lipoprotein(a); HbA1c = Hemoglobin A1C ; Free T3= Free triiodothyronine; Free T4= Free Thyroxine ; TT3= Total triiodothyronine; TT4= Total Thyroxine; TSH = Thyroid-Stimulating Hormone; LM = left main; RCA = right coronary artery; LAD = left anterior descending; LCX = left circumflex artery; CCB = Calcium channel blockers.

TableS2. Univariate analysis of predictors of MACE (excluding those loss to follow-up)

Variable	HR (95%CI)	P value
Age (per one year increase)	1.057 (1.034, 1.080)	<.0001
Sex (female as reference)	1.490 (0.931, 2.385)	0.0965
AMI (no as reference)	1.548 (0.487, 4.927)	0.4590
Hypertension (no as reference)	1.465 (0.927, 2.316)	0.1019
Diabetes (no as reference)	1.415 (0.905, 2.212)	0.1281
Hyperlipidemia (no as reference)	0.877 (0.583, 1.318)	0.5266
Smoke (no as reference)	1.705 (1.134, 2.564)	0.0103
Alcohol (no as reference)	1.336 (0.891, 2.002)	0.1610
BMI (per one kg/m^2 increase)	0.950 (0.890, 1.015)	0.1276
Heart rate (per one bpm increase)	0.999 (0.981, 1.018)	0.9575
Systolic blood pressure (per one mmHg increase)	1.013 (1.001, 1.025)	0.0329
LVEF (per 1% increase)	0.965 (0.934, 0.996)	0.0291
NT-proBNP (per one doubling increase)	1.659 (1.268, 2.170)	0.0002
TNI (per one unit increase)	0.995 (0.962, 1.030)	0.7928
HsCRP (per one mg/L increase)	1.065 (1.015, 1.119)	0.0109
Creatine (per one umol/L increase)	1.000 (0.998, 1.002)	0.8042
D-Dimer (per one ug/ml increase)	1.069 (0.887, 1.287)	0.4847
Endothelin (per one pmol/L increase)	1.901 (1.081, 3.344)	0.0258
ESR (per one mm/h increase)	1.014 (0.995, 1.033)	0.1572
NEU (per one percentage increase)	0.999 (0.977, 1.023)	0.9611
HbA1c (per one percentage increase)	1.204 (1.039, 1.396)	0.0137
Fasting glucose (per one mg/L increase)	1.026 (0.934, 1.127)	0.5915
TC (per one mmol/L increase)	0.885 (0.727, 1.079)	0.2270
LDL-c (per one mmol/L increase)	0.916 (0.725, 1.158)	0.4626
HDL-c (per one mmol/L increase)	0.768 (0.389, 1.518)	0.4486
Lpa (per one mg/L increase)	1.000 (0.999, 1.001)	0.7201
Free T_3 (per one pg/mL increase)	0.459 (0.275, 0.768)	0.0030
Free T_4 (per one pg/ml increase)	1.615 (0.474, 5.502)	0.4438
TT3 (per one ng/mL increase)	0.634 (0.252, 1.594)	0.3326
TT4 (per one ug/mL increase)	1.056 (0.943, 1.183)	0.3461
TSH (per one uIU/mL increase)	1.002 (0.958, 1.047)	0.9422
LM (no as reference)	1.611 (0.654, 3.965)	0.2998
RCA (no as reference)	1.082 (0.671, 1.744)	0.7471
LAD (no as reference)	0.822 (0.544, 1.241)	0.3501
LCX (no as reference)	1.457 (0.954, 2.225)	0.0817
Triple-vessel disease	1.091 (0.401, 2.970)	0.8651

AMI = acute myocardial infarction; BMI = body mass index; LVEF = left ventricular ejection fraction; TNI = troponin; hsCRP=high-sensitivity C-reactive protein; ESR = erythrocyte sedimentation rate; NEU = Neutrophil; HbA1c
= Hemoglobin A1C; TC = Total cholesterol; LDL-c = low-density lipoprotein cholesterol; HDL-c = high-density lipoprotein cholesterol; Lpa = Lipoprotein(a); Free T3= Free triiodothyronine; Free T4= Free Thyroxine ; TT3= Total triiodothyronine; TT4= Total Thyroxine; TSH = Thyroid-Stimulating Hormone; LM = left main; RCA = right coronary artery; LAD = left anterior descending; LCX = left circumflex artery.

3 **Table S3. Collinearity analysis of included variables in regression models with NT-proBNP.**

	Unstandardized coefficients		Standardized coefficients	t	Sig.	Collinearity Statistics	
	B	Std.error	Beta			Tolerance	VIF
Constant	9.903	0.392		25.255	0.000		
Age	0.008	0.002	0.108	3.621	0.000	0.849	1.178
Sex	0.051	0.051	0.035	1.014	0.311	0.640	1.562
BMI	-0.006	0.006	-0.030	-1.029	0.304	0.916	1.092
Smoke	0.007	0.046	0.005	0.163	0.870	0.695	1.439
Hypertension	-0.060	0.041	-0.041	-1.450	0.147	0.929	1.076
Diabetes	0.015	0.057	0.009	0.272	0.786	0.633	1.579
EF	-0.018	0.004	-0.137	-4.948	0.000	0.990	1.010
hsCRP	0.030	0.006	0.137	4.803	0.000	0.936	1.069
Endothelin	0.623	0.085	0.204	7.307	0.000	0.967	1.034
HbA1c	-0.020	0.024	-0.029	-0.840	0.401	0.636	1.573
FT3	0.006	0.058	0.003	0.106	0.915	0.847	1.180

Supplemental Figure 1. Relationship between NT-proBNP and creatine(A) or ejection fraction (B)

A.

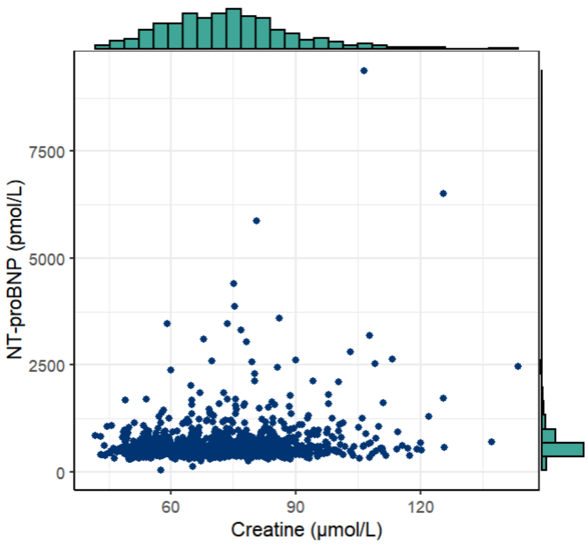

B.

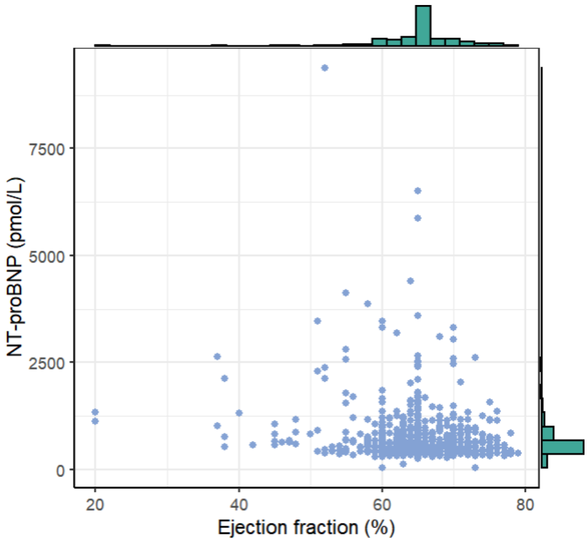

5 **Supplemental Figure 2.** Schoenfeld individual test for each covariate included in cox regression model. NT-proBNP was modelled as a continuous variable (A) or categorical variable (B).

A.

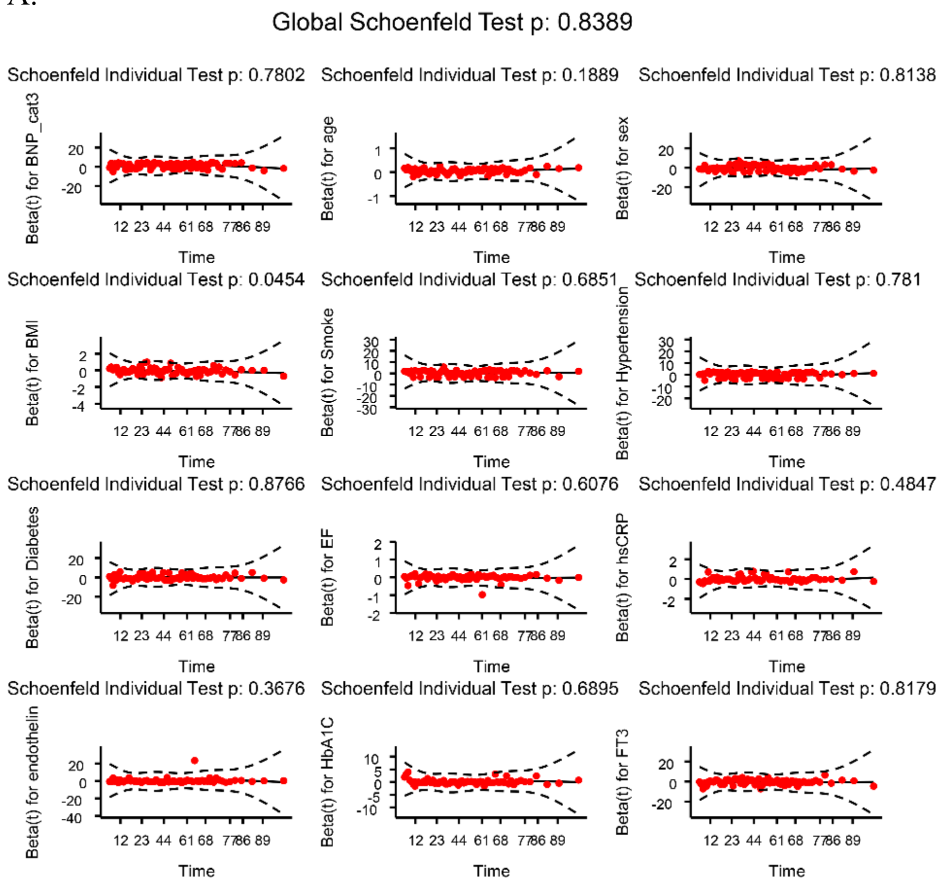

B.

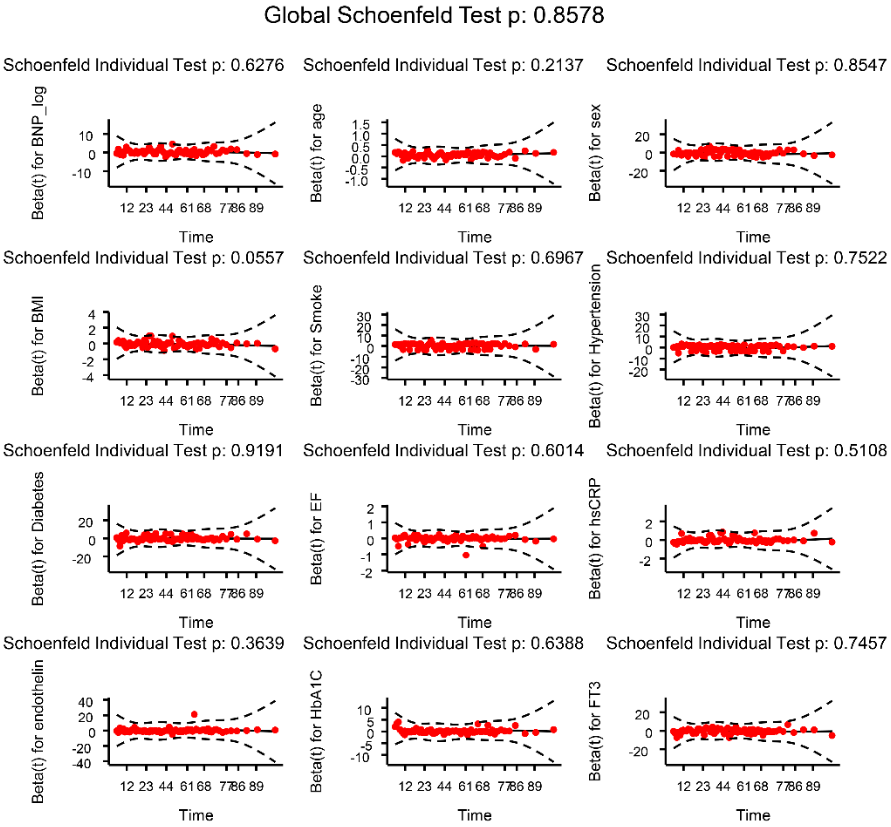
Global Schoenfeld Test p: 0.8578
Schoenfeld Individual Test p: 0.6276
Schoenfeld Individual Test p: 0.2137
Schoenfeld Individual Test p: 0.8547
Beta(t) for BNP_log
Beta(t) for age
Beta(t) for sex
Schoenfeld Individual Test p: 0.0557
Schoenfeld Individual Test p: 0.6967
Schoenfeld Individual Test p: 0.7522
Beta(t) for BMI
Beta(t) for Smoke
Beta(t) for Hypertension
Schoenfeld Individual Test p: 0.9191
Schoenfeld Individual Test p: 0.6014
Schoenfeld Individual Test p: 0.5108
Beta(t) for Diabetes
Beta(t) for EF
Beta(t) for hsCRP
Schoenfeld Individual Test p: 0.3639
Schoenfeld Individual Test p: 0.6388
Schoenfeld Individual Test p: 0.7457
Beta(t) for endothelin
Beta(t) for HbA1C
Beta(t) for FT3
Time
